# Immunoinflammatory Profile of FGF‐18, IL‐35 and Glutamic Acid Decarboxylase in Patients With Diabetic Foot Ulcers

**DOI:** 10.1111/iwj.70990

**Published:** 2026-07-03

**Authors:** Hemin Mohamad Hussein, Ahmed Alkhuzai, Shukur Wasman Smail, Taha Othman Mahwi, Jonas Bystrom, Christer Janson, Kawa Amin

**Affiliations:** ^1^ Department of Biology, College of Science University of Sulaimani Sulaymaniyah Iraq; ^2^ Department of Clinical Sciences, College of Medicine University of Sulaimani Sulaymaniyah Iraq; ^3^ College of Pharmacy Cihan University‐Erbil Erbil Kurdistan Region Iraq; ^4^ Department of Biology, College of Science Salahaddin University‐Erbil Erbil Kurdistan Region Iraq; ^5^ Department of Medical Science, Respiratory Allergy and Sleep Research Uppsala University Uppsala Sweden; ^6^ Centre for Translational Medicine and Therapeutics, William Harvey Research Institute Queen Mary University of London London UK; ^7^ Department of Basic Medical Science, College of Medicine University of Sulaimani Sulaymaniyah Iraq

**Keywords:** diabetic foot ulcer, fibroblast growth factor‐18, glutamic acid decarboxylase, IL‐35, inflammation, type 2 diabetes

## Abstract

Diabetic foot ulcer (DFU) is a serious problem that may cause amputation of the lower extremities in patients with diabetes. The present research aimed to assess the localised tissue expression and potential immunoinflammatory crosstalk among fibroblast growth factor‐18 (FGF‐18), glutamic acid decarboxylase (GAD) and interleukin‐35 (IL‐35) in DFU compared to non‐diabetic controls (NDCs), while also examining the systemic serum levels of FGF‐18 and IL‐35. Venous blood was collected from 80 patients with DFU and 100 NDC, and the concentrations of serum IL‐35, FGF‐18 and blood haemoglobin A1c (HbA1c) were analysed. Aseptically collected biopsy samples were obtained from FUs of 30 type 2 diabetes (T2D) patients and from accidental foot wounds of 30 NDC. Biopsies were preserved in formalin (10%) until paraffin blocks were prepared. The immunohistochemical methodology used antibodies specifically to identify tissue FGF‐18, GAD and IL‐35 in the soft tissue specimens. The tissue expression of FGF‐18, IL‐35 and GAD was significantly higher in DFU compared to NDC (*p* ≤ 0.0001), suggesting a strong localised immunoinflammatory role, whereas serum levels of FGF‐18 and IL‐35 remained statistically unchanged. Furthermore, significant positive correlations observed between tissue IL‐35 and FGF‐18 (*r* = 0.67, *p* ≤ 0.05) and between tissue IL‐35 and GAD (*r* = 0.60, *p* ≤ 0.05) indicate robust immunoinflammatory crosstalk. The marked and correlated elevation of FGF‐18, IL‐35 and GAD specifically within DFU tissue, without changes in serum levels of FGF‐18 and IL‐35, establishes a robust, compartmentalised immunoinflammatory axis that drives chronic pathology and presents novel targets for localised therapeutic intervention.

## Key Points

- Diabetic foot ulcer tissue showed significantly increased expression of FGF‐18, IL‐35, and GAD compared with non‐diabetic control tissue.
- Serum levels of FGF‐18 and IL‐35 were not significantly different between diabetic foot ulcer patients and controls, suggesting a localized tissue‐specific response.
- Tissue IL‐35 was positively correlated with both FGF‐18 and GAD, indicating possible immunoinflammatory crosstalk within diabetic foot ulcer lesions.
- These findings highlight FGF‐18, IL‐35, and GAD as potential localized biomarkers and therapeutic targets in diabetic foot ulcer pathology.

## 1 Introduction

Foot ulcers (FUs) in type 2 diabetes (T2D) patients are a severe disease that may result in the amputation of lower extremities [1]. Diabetic foot ulcer (DFU) is a prevalent consequence of diabetes that can significantly affect the bones in the feet. Early symptoms of DFUs include atypical oedema, exudate, maceration, erythema, stinging, irritation and odour. The most evident symptom of severe DFU is a black material known as eschar encircling the ulcer area [2].

Individuals with T2D often get infections because of DFU. FUs in T2D patients are a major global cause of non‐traumatic lower extremity amputations. Approximately 85% of lower limb amputations result from DFU [1, 3]. The predominant risk factors for DFU are peripheral artery occlusive disease, diabetic peripheral neuropathy and physical foot abnormalities. The predominant cause of hospital admissions among diabetic patients is FUs [4, 5, 6].

The incidence of DFU varies significantly based on the demographic and study methodology [7, 8]. Recent data about the comparative incidence of T1D and T2D are conflicting [7, 9], and variations in age and diabetes duration will significantly affect DFU risk among groups with T1D and T2D. The International Diabetes Federation (IDF) indicated that a survey conducted in 2021 found the number of diabetic individuals was 537 million in the age range (20–79) [10]. Diabetic patients are more susceptible to injuries and ulcers because of a lack of defensive sensation in their lower limbs. This loss of sensation results from the upregulation of aldose reductase and sorbitol dehydrogenase in response to high glucose levels in their blood, leading to an elevated synthesis of fructose and sorbitol [11].

Several molecular dysfunctions influence the transition from hyperglycaemia to DFU in the process of wound healing. Haemostasis, proliferation, inflammation and remodelling are standard physiological processes involved in wound healing. Acute wounds advance sequentially through these stages, but chronic non‐healing DFU remain stationary in single or several phases. Neutrophils generally generate granular substances in their granules to eliminate attacking infectious agents during the early phases of the repair stage [12, 13]. In diabetes, the neuroendocrine system becomes dysregulated, leading to an overproduction of cytokines and superoxide, which initiates a pro‐inflammatory cascade that interferes with wound healing [14, 15].

Fibroblast growth factor (FGF)‐17 and FGF‐18 are two members of the FGF‐8 subfamily. These growth factors exhibit distinct expression patterns during embryonic development, which can be attributed to similarities in the amino acid sequences [16]. Glutamic acid decarboxylase 65 (GAD65) is an enzyme expressed not only in pancreatic β‐cells but also in neuronal and fibroblast lineages, including dermal, gingival and periodontal fibroblasts, where it catalyses the conversion of glutamate to γ‐aminobutyric acid (GABA) [17, 18]. Autoantibodies directed against GAD65 are well‐established biomarkers of autoimmune β‐cell destruction in type 1 diabetes and in latent autoimmune diabetes in adults (LADA), indicating a loss of immune tolerance towards β‐cell antigens [18]. Recent studies have revealed that a subset of patients clinically diagnosed with T2D also exhibit low‐titre GAD positivity, a condition associated with accelerated β‐cell decline, poorer glycaemic control and an earlier requirement for insulin therapy [19, 20].

The interleukin (IL)‐35 is made up of two portions: the IL‐12α (also called IL‐12p35) chain and the Epstein–Barr virus‐induced 3 (EBI3) chain [21, 22]. The IL‐12α part of IL‐35 is also present in IL‐12 (comprising IL‐12α and IL‐12β), but the other constituent of IL‐35, EBI3, is found in IL‐27 (which consists of EBI3 and IL‐27p28) [23]. IL‐35 is predominantly produced by regulatory T cells (Treg), regulatory B cells (Breg) and antigen‐presenting cells that express major histocompatibility complex II (MHCII). It is a distinct category of cytokine that can suppress or decrease the immune response and reduce inflammation. IL‐35 stimulates various T helper (Th) cell and memory T cell populations, resulting in distinct cytokine responses, crucial for the development of inflammatory and autoimmune disorders [24]. Prior research has demonstrated that IL‐35 predominantly promotes the development of Treg and Breg. Macrophages and effector T lymphocytes, Th1 and Th17, are suppressed by the cytokine [23].

FGF‐18, IL‐35 and GAD were selected as biomarkers in T2D with DFU because they capture complementary aspects of tissue remodelling, immune regulation and diabetes‐associated inflammation. FGF‐18 is implicated in skeletal and tissue repair processes relevant to chronic foot wounds [25, 26]. GAD65 is expressed in pancreatic β‐cells and several fibroblast lineages, and GAD autoimmunity in T2D has been linked to worse glycaemic control and faster β‐cell decline in a subset of patients [27, 28]. IL‐35 is an anti‐inflammatory cytokine of the IL‐12 family, produced mainly by Treg cells, and it has immunomodulatory effects through suppression of pro‐inflammatory cells and support of Treg/Breg‐associated regulation [29]. Taken together, these markers may reflect a shared immunoinflammatory axis in diabetic tissues; however, their local expression and roles in DFU remain poorly defined, which justifies examining both systemic levels and tissue expression in diabetic versus non‐diabetic control (NDC) foot wounds.

## 2 Materials and Methods

### 2.1 Study Population

The study was performed in accordance with the World Medical Association's Code of Ethics (Declaration of Helsinki) for studies involving human beings. The research protocol (No. 75, Date: 18 May 2024) was authorised by the Ethical Committee of the Sulaimani University, College of Medicine. Prior to their participation in the study, each subject provided written informed permission.

In this study, 80 individuals with T2D with DFU were enrolled between January 2025 and March 2025.

Participants were recruited following admission to medical facilities in Sulaymaniyah City, including Shar Hospital, the Diabetes and Endocrine Centre and private healthcare sectors. A specialist physician confirmed the diagnosis of T2D according to established diabetes diagnostic criteria [30]. For the blood‐sample analysis, the DFU group was compared with 100 NDCs. NDC inclusion criteria were: adults without a history of diabetes, no clinical evidence of peripheral vascular disease or neuropathy and no current acute or chronic inflammatory, autoimmune or malignant disease. Pregnancy, smoking, alcoholism, other autoimmune diseases and steroid use were exclusionary criteria for all participants. Patients who met any of the exclusion criteria were excluded. The overall sample size was estimated a priori using G*Power software based on an a priori power analysis, using an expected effect size (Cohen method) to provide adequate power for detecting group differences [31].

Biopsies were obtained from the FU of 30 out of the 80 T2D patients. Additionally, 30 biopsy samples were collected from NDCs who presented with accidental cuts or fractures of the foot at the Emergency Hospital in Sulaymaniyah, Iraq. Biopsy collection was performed in this subgroup of 30 DFU patients and 30 NDCs because tissue sampling required surgical/clinical availability and written informed consent. This subgroup size was considered adequate for immunohistochemical comparison and was constrained by ethical and practical feasibility.

### 2.2 Blood Samples

Venipuncture was performed to obtain blood samples in two test tubes: one containing EDTA for whole blood and another containing gel and clot activator for serum. Haemoglobin A1c (HbA1c) was performed using whole blood and COBAS C111 (Roche). To separate the serum from the clotted portion, the serum tubes were centrifuged for 10 min at 5000 rpm after being placed on the rack upright for 15 min without shaking, allowing for blood clotting. Serum specimens were separated into two portions: one portion was utilised to perform numerous tests, including triglycerides, total cholesterol, low‐density lipoprotein (LDL) cholesterol, high‐density lipoprotein (HDL) cholesterol and fasting blood glucose (FBG). Three aliquots were prepared from the second portion of the serum, and the aliquots were kept at −70°C until they were studied using the enzyme‐linked immunosorbent assay (ELISA) for two biological markers (FGF‐18 and IL‐35).

### 2.3 Qualitative and Quantitative Analysis of FGF‐18 and IL‐35

An ELISA kit that was specifically designed for each biomarker was used. ELISA for FGF‐18 (Cat No. E5287Hu) and IL‐35 (Cat No. E0042Hu). After thoroughly thawing the serum samples, the ELISA kits were allowed to remain at room temperature for 30 min, in accordance with the manufacturer's instructions. To create standards one through five, the standard solution from each kit was diluted to the desired concentration. Following the assay protocol, all standards were examined step‐by‐step in conjunction with serum samples. The optical density of each ELISA plate was measured using a Biotek ELX800 microplate reader. Prism 9.0 software (GraphPad) was used to determine the levels of biomarkers separately in specific ELISA kits by creating a standard curve with known quantities of standards and measuring the absorbance of each.

### 2.4 Biopsy Samples

The experienced physician aseptically obtained biopsy specimens from the lower extremities of participants. Biopsies were collected in tubes with 10% Formalin and subsequently chopped for block preparation. Soft tissues were placed in containers, and processing was conducted using the Hito‐Tek VP1TM (SAKURA) machine, a fully automated system. A Tissue‐Tek TEC TM embedding machine (SAKURA) was used for the preparation of paraffin blocks.

### 2.5 Section Preparation for Immunohistochemistry

A rotary microtome machine (Accu‐CUT SRM, SAKURA) was used to produce thin sections (5 μm). These thin sections were then placed onto slides by using a water bath (Cell Path, UK) at 40°C. Charged slides for the use of antibodies and regular slides for the use of eosin and haematoxylin were used. Deparaffinisation of biopsy sections was performed by heating the sections at 75°C for 45 min using an oven machine (Acculab, USA). Subsequently, haematoxylin and eosin (HE) were used to stain the tissue samples, and for this procedure, an automated machine (Tissue‐Tek Prisma Plus, SAKURA) was used.

### 2.6 Immunohistochemical Staining Protocol

Tissue sections after formalin fixation and paraffin embedding were placed on silanised microscope slides (DAKO, USA) after being cut to a thickness of 5 μm. Slides were dried by heating at 70°C for 1 h in an oven (Acculab, USA). Deparaffinisation of all sections was performed using xylene, followed by rehydration through a sequence of ethanol concentrations in order. The slides with sections were heated at 70°C for 1 h in an oven (Acculab, USA) for deparaffinisation. Antigen retrieval of all slides was performed using an Envision Target retrieval solution (DAKO, USA) and a PT Module machine under the following conditions: pH nine and heating for 40 min at 100°C.

Subsequently, phosphate‐buffered saline (PBS) at pH 7.2 was used to wash the slides twice (3 min each). A pen (Immerges) (Vector Laboratories) was used to create a circle around each section. A peroxidase blocker (Bio SB, California, USA) was used to deactivate endogenous peroxidase, and sections were incubated with 200 μL for 10 min at 25°C. Subsequently, two more washing steps were performed with PBS at pH 7.2 (DAKO, USA) to eliminate the peroxidase inhibitor.

Primary antibodies were purchased as concentrated, while based on the guidelines in the kit insert they were diluted in the lab by using an antibody diluent (Bio SB, California, USA): polyclonal antibodies against IL‐35 derived from rabbit (BT‐AP04435, BioScience, China) at a 1:100 dilution, polyclonal antibodies against FGF‐18 derived from rabbit (E‐AB‐15030, BT Lab, China) at a 1:100 dilution, and polyclonal antibodies against GAD derived from rabbit (E‐AB‐15661, BT Lab, China) at a 1:100 dilution. Polyclonal specific antibodies derived from rabbit (50 μL) were applied to the sections individually and incubated for 45 min at room temperature. Subsequently, two more washing steps were performed with PBS at pH 7.2 (DAKO, USA). Additionally, slices of biopsy specimens were treated with 50 μL of enzyme conjugates (goat anti‐rabbit antibody (derived from goat) + horseradish peroxidase) as secondary antibody and enzyme (K8002, DAKO, USA) and incubated at room temperature for 45 min. Subsequently, the slides were washed for 3 min (2×) in PBS at pH 7.2 (DAKO, USA).

The stock solution of chromogen (DAB (diaminobenzidine) and chromogen) was diluted to a ratio of 1:20 according to established protocols (K8002, DAKO, USA). Subsequently, one to two drops of the diluted chromogen were applied to the sections, and sections were incubated for 5 min at room temperature. All tissue sections were washed with distilled water to eliminate extra chromogen. Haematoxylin was used for 1 min to stain the slides, and subsequently, the slides were rinsed with distilled water. After that, the slides were immersed for 1 min in each of the graded concentrations of ethanol separately and in order (70%, 90% and finally 100% ethanol). Afterward, all slides were soaked in xylene for 1 min, followed by the application of a mounting medium (DAKO, USA) and coverslips. After air‐drying of the slides, the sections were examined using a light microscope (Leica, Germany).

### 2.7 Qualitative and Quantitative Analysis of Stained Tissue Sections

Slides with specimens were labelled, and from each section, three different slides were prepared. Slides were used to examine the production of GAD, FGF‐18 and IL‐35. Specific antibodies, including anti‐GAD, anti‐FGF‐18 and anti‐IL‐35, were used accordingly. A light microscope (Leica, Germany) was used to examine slides at different magnification powers (10×, 20×, 40× and 60×). The microscope was used to quantify the overall area of the stained section, which included both stained and unstained cells. A microsystem digital camera (DC 300F) was employed to record and transmit images to a computer. The assessment and quantification of human cells expressing IL‐35, FGF‐18 and GAD was conducted using Qwin version 2.7 software. The quantity of cells expressing IL‐35, FGF‐18, and GAD per square millimetre was quantified. Subsequently, statistical analysis was performed.

### 2.8 Statistical Analysis

Statistical analyses were performed using GraphPad Prism version 9.0. The data met the assumptions of normality (D'Agostino, Shapiro–Wilk tests) and homogeneity of variances (Levene's test); therefore, results are presented as mean ± standard deviation (SD). Parametric tests were applied, including one‐way analysis of variance (ANOVA) for comparisons among more than two groups and the independent *t*‐test for comparisons between two groups. Pearson's correlation analysis was used to evaluate associations between biomarkers and clinical variables such as diabetes duration, age, and body mass index (BMI). A *p* < 0.05 was considered statistically significant.

## 3 Results

### 3.1 General Characteristics of Patients With DFU

Table 1 summarises the baseline characteristics of the study population. Male sex was comparable between the DFU group and the NDC group [50/80 (62.5%) vs. 60/100 (60.0%), respectively]. The mean age of patients with DFU was significantly higher than that of controls (59.67 ± 9.78 vs. 50.04 ± 11.07 years, *p* = 0.0023).

**TABLE 1:** Characteristics of study participants with T2D with DFU compared to non‐diabetic controls.

Parameters	NDC (*N* = 100)	DFU (*N* = 80)	*p*
M (*N*, %)	(60, 60%)	(50, 62.5%)	—
F (*N*, %)	(40, 40%)	(30, 37.5%)	—
Age (years) (mean ± SD)	50.04 ± 11.07	59.67 ± 9.78	0.0023
HbA1c% (mean ± SD)	5.41 ± 0.37	8.92 ± 2.57	< 0.0001
BMI	28.35 ± 3.58	27.03 ± 4.24	0.1815

*Note:* Data are presented as number and percentage [*n* (%)] for categorical variables and as mean ± standard deviation (SD) for continuous variables. The independent‐samples *t*‐test was used to compare continuous variables between groups. A *p* < 0.05 was considered statistically significant.

Abbreviations: BMI, body mass index; DFU, diabetic foot ulcer; F, female; HbA1c, glycated haemoglobin; M, male; NDC, non‐diabetic controls; SD, standard deviation; T2D, type 2 diabetes mellitus.

### 3.2 Comparison of Serum Concentrations of FGF‐18 and IL‐35 Between Non‐Diabetic Controls and T2D Patients With DFU

The serum level of FGF‐18 was raised in T2D with DFU in comparison with NDC; however, the difference was not statistically significant (*p* = 0.6177), as presented in Table 2. The serum level of IL‐35 was elevated in T2D with DFU compared to NDC; nevertheless, the variance was not statistically significant (*p* = 0.1358).

**TABLE 2:** Comparison of the serum concentrations of FGF‐18 and IL‐35 between NDC and T2D with DFU.

Biomarkers	NDC (*N* = 100) mean ± SD	DFU (*N* = 80) mean ± SD	*p*
FGF‐18 (ng/L)	271.1 ± 69.56	279.4 ± 56.87	0.6177
IL‐35 (ng/mL)	2.87 ± 1.06	3.32 ± 1.04	0.1358

*Note:* Data are presented as mean ± standard deviation (SD). Serum biomarker concentrations were compared between groups using the independent‐samples *t*‐test. A *p* < 0.05 was considered statistically significant.

Abbreviations: DFU, diabetic foot ulcer; FGF‐18, fibroblast growth factor 18; IL‐35, interleukin‐35; NDC, non‐diabetic controls; SD, standard deviation; T2D, type 2 diabetes mellitus.

### 3.3 Comparing Tissue GAD, FGF‐18 and IL‐35 Expression Between the Groups, in Blood and Tissue Samples

As summarised in Table 3, the quantity of cells expressing FGF‐18 decreased significantly in NDC compared to T2D patients with DFU (*p* ≤ 0.0001). The quantity of cells producing FGF‐18 is significantly increased in biopsy sections of DFU in comparison with NDC.

**TABLE 3:** Comparison of the number of biomarker‐producing cells between non‐diabetic controls and T2D patients with DFU, as assessed by immunohistochemistry.

Biomarkers	NDC (*N* = 30) mean ± SD	DFU (*N* = 30) mean ± SD	*p*
FGF‐18 (positive cells per mm^2^)	78 ± 81.5	778 ± 163.7	< 0.0001
GAD (positive cells per mm^2^)	45 ± 22.7	645 ± 517.2	< 0.0001
IL‐35 (positive cells per mm^2^)	66 ± 65.78	299 ± 147.8	< 0.0001

*Note:* Data are presented as mean ± standard deviation (SD) and expressed as positive cells per mm^2^. Comparisons between groups were performed using the independent‐samples *t*‐test. A *p* < 0.05 was considered statistically significant.

Abbreviations: DFU, diabetic foot ulcer; FGF‐18, fibroblast growth factor 18; GAD, glutamic acid decarboxylase; IHC, immunohistochemistry; IL‐35, interleukin‐35; NDC, non‐diabetic controls; SD, standard deviation; T2D, type 2 diabetes mellitus.

**FIGURE 1:**
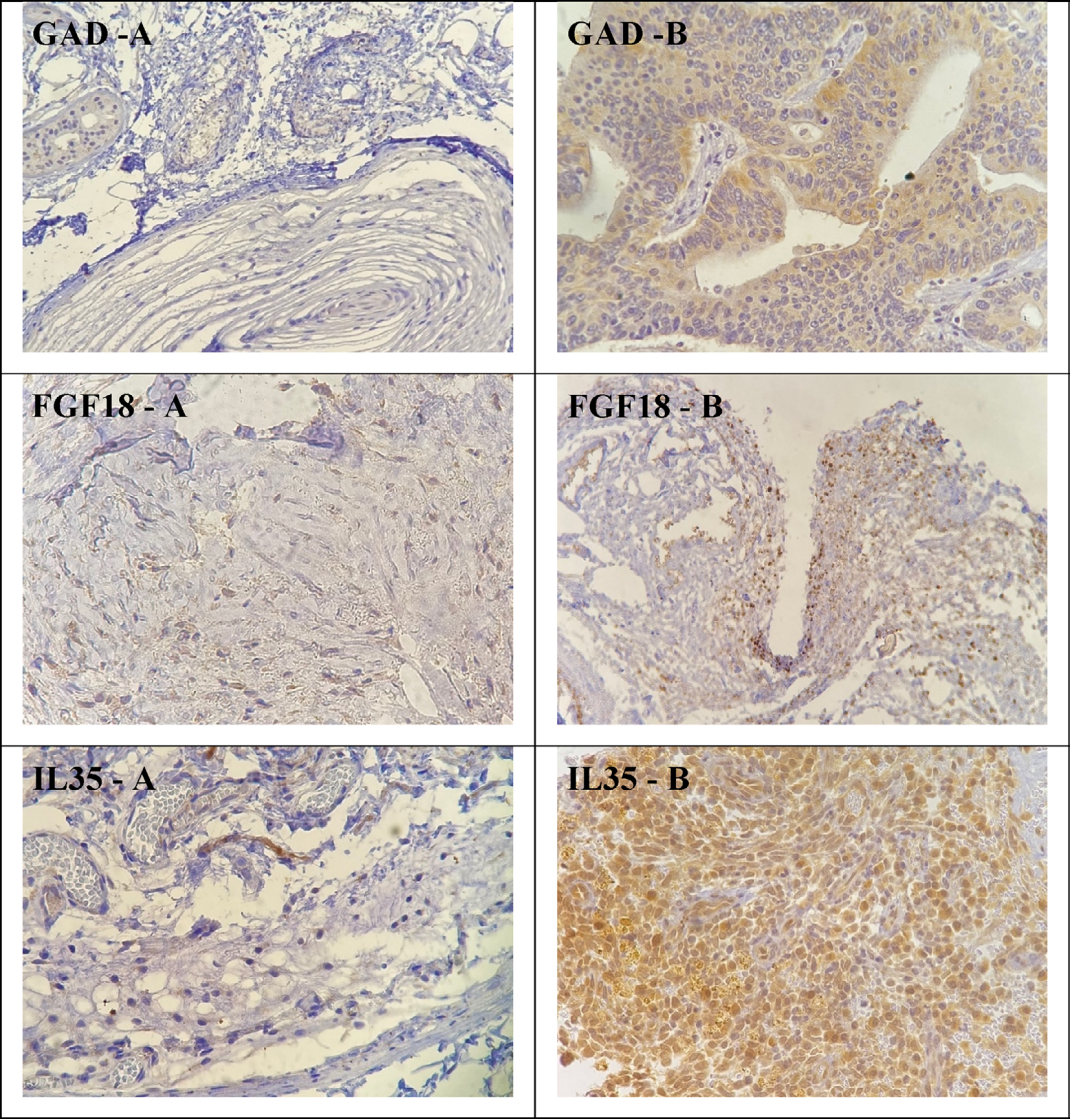
Immunohistochemical examination of GAD, FGF‐18 and IL‐35 production in foot biopsies. (A) Production of GAD, FGF‐18 and IL‐35 from cells in NDC foot biopsies (40×). (B) Production of GAD, FGF‐18 and IL‐35 from cells in foot biopsies from T2D patients with DFU (40×). DFU, diabetic foot ulcer; FGF‐18, fibroblast growth factor 18; GAD, glutamic acid decarboxylase; IL‐35, interleukin‐35; NDC, non‐diabetic controls; T2D, type 2 diabetes mellitus.

A significantly increased quantity of cells expressing GAD was seen in T2D patients with DFU in comparison with NDC (*p* ≤ 0.0001), as outlined in Table 3. The expression of GAD in the tissue samples from T2D patients with DFU was elevated compared to the GAD expression in the soft tissue sections from NDC (Figure 1). The number of positive cells expressing IL‐35 was markedly reduced in NDC compared to T2D patients with DFU (*p* ≤ 0.0001), as illustrated in Table 3. IL‐35 expression is elevated in the tissue samples from T2D patients with DFU compared to that in tissue specimens from NDC (Figure 1).

As seen in Figure 1, an elevated quantity of FGF‐18 producing cells has been recorded in the biopsy samples of T2D patients with DFU compared to the amount of FGF‐18 producing cells in biopsy samples from NDCs. Figure 1 displays an elevated amount of IL‐35 production in the tissue samples from T2D patients with DFU compared to the production of IL‐35 in tissue samples from NDCs.

**FIGURE 2:**
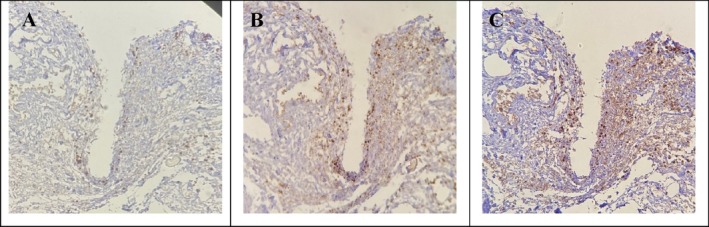
The production of GAD, FGF‐18 and IL‐35 was compared by immunohistochemical analysis. The tissue specimen was collected from a T2D patient with DFU. (A) GAD production was studied by using anti‐GAD. (B) FGF‐18 production was studied using anti‐FGF‐18. (C) IL‐35 production was studied using anti‐IL‐35. DFU, diabetic foot ulcer; FGF‐18, fibroblast growth factor 18; GAD, glutamic acid decarboxylase; IL‐35, interleukin‐35; T2D, type 2 diabetes mellitus.

As shown in Figure 2, representative staining from the same DFU tissue region demonstrated variable expression of these markers. The number of IL‐35‐positive cells was higher than that of FGF‐18‐positive and GAD‐positive cells. In addition, FGF‐18 expression was higher than GAD expression based on the number of positively stained cells. Because the same tissue biopsy region was used for comparison, these findings provide a descriptive overview of the relative expression pattern of GAD, FGF‐18 and IL‐35 in DFU tissue.

### 3.4 Correlation Among Tissue Biomarkers in T2D With DFU

**FIGURE 3:**
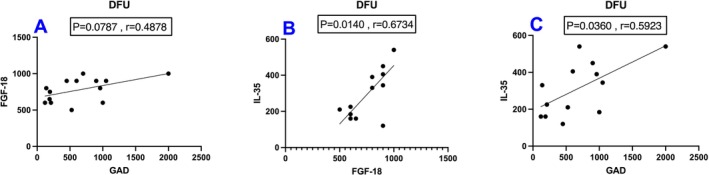
Pearson correlation between the quantity of positive cells that produce GAD, FGF‐18 and IL‐35 in T2D with DFU. (A) Correlation between the number of cells and the production of GAD and FGF‐18. (B) Correlation between the number of cells based on the output of FGF‐18 and IL‐35. (C) Correlation between the quantity of cells based on the production of GAD and IL‐35. DFU, diabetic foot ulcer; FGF‐18, fibroblast growth factor 18; GAD, glutamic acid decarboxylase; IL‐35, interleukin‐35; T2D, type 2 diabetes mellitus.

As illustrated in Figure 3 and Figure S1, in T2D patients with DFU, no significant correlation was discovered between tissue FGF‐18 and tissue GAD (*p* = 0.0787, *r* = 0.49) (CI [−0.08 to 0.81]). However, between tissue IL‐35 and tissue FGF‐18, a significant positive correlation was observed (*p* = 0.0140, *r* = 0.67) (CI [0.18–0.9]). A significant positive correlation was observed between tissue GAD and tissue IL‐35 (*p* = 0.0360, *r* = 0.60) (CI [0.04–0.7]).

**FIGURE 4:**
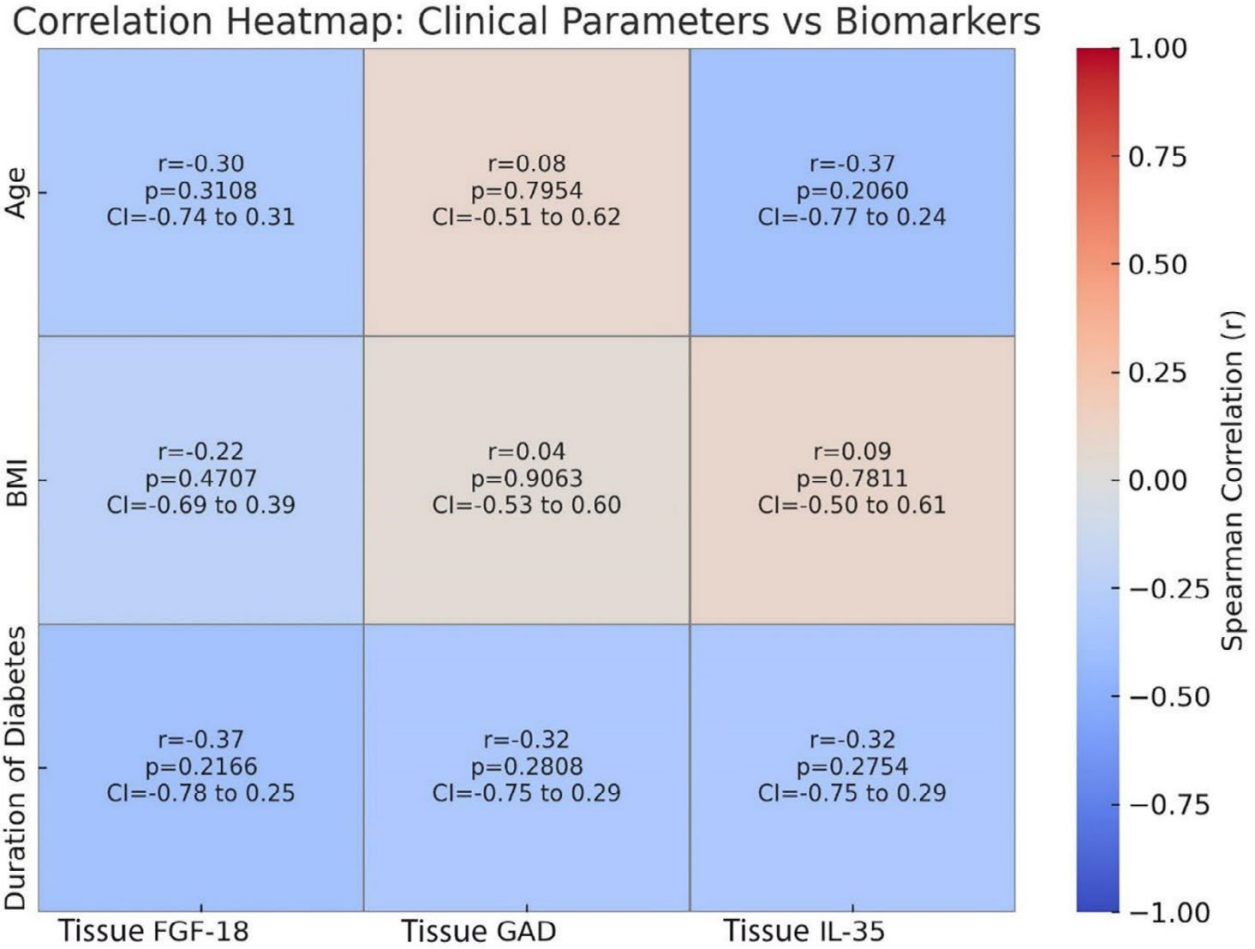
Correlation heatmap showing relationships between clinical parameters (age, body mass index [BMI] and duration of diabetes) and biomarker‐positive cells (FGF‐18, GAD, IL‐35). Spearman correlation was used to calculate correlation coefficients (*r*), *p* values and 95% confidence intervals (CI). BMI, body mass index; CI, confidence interval; FGF‐18, fibroblast growth factor 18; GAD, glutamic acid decarboxylase; IL‐35, interleukin‐35.

As presents in Figure 4, there were no significant associations between the age of patients with DFU and tissue FGF‐18 (*p* = 0.3108, *r* = −0.3) (CI [−0.74 to 0.31]), GAD (*p* = 0.7954, *r* = 0.08) (CI [−0.51 to 0.62]) and tissue IL‐35 (*p* = 0.2060, *r* = −0.37) (CI [−0.77 to 0.24]). Similarly, no significant association was observed between BMI and tissue FGF‐18 (*p* = 0.4707, *r* = −0.22) (CI [0.69–0.39]), tissue GAD (*p* = 0.9063, *r* = 0.04) (CI [−0.53 to 0.6]) and tissue IL‐35 (*p* = 0.7811, *r* = 0.09) (CI [−0.50 to 0.61]). The association between the tissue FGF‐18 and duration of having diabetes (*p* = 0.2166, *r* = −0.37) (CI [−0.78 to 0.25]), GAD (*p* = 0.2808, *r* = −0.32) (CI [−0.75 to 0.29]) and tissue IL‐35 (*p* = 0.2754, *r* = −0.32) (CI [−0.75 to 0.29]) was not statistically significant. Furthermore, Pearson correlation analysis showed no significant correlation between HbA1c and IL‐35 positive producing cells (*r* = −0.325, *p* = 0.237), GAD positive producing cells (*r* = −0.181, *p* = 0.518) or FGF‐18 positive producing cells (*r* = −0.058, *p* = 0.838) in the studied patients (*n* = 15). These results indicate no significant association between HbA1c levels and the assessed markers (Figure S2).

## 4 Discussion

Tissue expression of positive cells for FGF‐18, IL‐35 and GAD was significantly higher in DFU compared to controls. Conversely, serum levels of FGF‐18 and IL‐35 showed no statistically significant difference. Concurrent with this finding, a recent study discovered no significant differences in the blood concentration of FGF‐18 between NDCs and T2D patients [25]. On the other hand, prior research found that T2D had a significantly higher serum level of FGF‐18 than control [32]. The FGF family has been investigated for its potential therapeutic benefits in DFU. Prior research indicates that FGF can promote angiogenesis and stimulate the growth of fibroblasts, a significant factor in angiogenesis [26]. Similarly, FGF‐18 levels have been investigated in different conditions and be elevated in breast cancer [33], ovarian cancer [34] and colon tumour [35]. Because it regulates metabolic pathways, FGF has demonstrated potential therapeutic effects in diabetes, controlling insulin resistance and enhancing homeostasis in adipose tissue [36].

Various categories of FGF have been identified, including aFGF, bFGF, and the FGF 15/19 subfamily, which demonstrate an impact on the wound healing process of diabetic wounds [26]. While FGF demonstrates potential therapeutic advantages in DFU, there is insufficient data regarding the specific function of FGF‐18 in this context. Furthermore, no direct evidence has been found to connect FGF‐18 to DFU in humans. This study on FGF‐18 in T2D patients with DFUs is novel. It is suggested that increased production of FGF‐18 contributes positively to the recovery process. Additional investigation is required to assess the possible curative advantages of FGF‐18 in the management of DFU.

The current research found a significantly greater number of positive cells expressing IL‐35 in DFU compared to NDC. Furthermore, in terms of IL‐35's systematic expression, T2D patients with DFUs had greater serum levels of IL‐35 than control. Information regarding IL‐35's function in DFUs is limited. T2D patients and NDCs did not show significant differences in their blood levels of IL‐35, according to a recent study [25]. IL‐35 suppresses inflammation and promotes tissue healing in various diseases due to its anti‐inflammatory properties. It has been shown to inhibit the synthesis of cytokines that promote inflammation, including tumour necrosis factor‐alpha (TNF‐α) and IL‐6, across multiple disorders [37].

T2D and HC did not significantly differ in their blood concentration levels of IL‐35, according to recent studies [38]. In line with our research, increased blood concentration of IL‐35 was recorded in patients with T1D [39]. Furthermore, the progression of T1D and T2D may be influenced by IL‐35. IL‐35 was thought to lower the production of inflammatory cytokines [40]. According to [41], IL‐35 has been demonstrated to control Treg suppression, prevent Th17 cell development, and thus minimise ongoing immunological damage to beta cells.

Pancreatic beta cells, human gingival fibroblasts, periodontal fibroblasts, cutaneous fibroblasts and mesangial cells express GAD65 [27, 28]. The present investigation identified a significantly increased number of positive cells expressing GAD in T2D patients with DFU compared to NDC (*p* < 0.0001). This indicates that GAD became highly produced during inflammation. Consistent with our findings, prior research has investigated GAD65 expression in normal and inflammatory tissues, revealing an overexpression of GAD65 in inflamed tissues. GAD65 was upregulated in mice subjected to inflammation induced by a single administration of lipopolysaccharide into the abdominal cavity [42]. In the rat nervous system, peripheral inflammation correlates with increased GAD immunogenicity [43].

Moreover, both GADA concentrations and GAD quantities can be influenced by inflammation [28]. Elevated GAD synthesis may result in an increased titre of GADA. In individuals with T2D and DFU, a significant positive association was observed between the quantity of cells expressing FGF‐18 and the quantity of cells expressing IL‐35. Additionally, a notable positive association was observed between the number of cells expressing IL‐35 and the number of cells expressing GAD. Similarly, a recent study identified a linear positive connection between GADs and various cytokines (FGF‐9, FGF‐18, DEL‐1, IL‐31 and IL‐35) in T2D [25]. However, in the present research, no significant correlation was found between the number of cells expressing FGF‐18 and the number of cells expressing GAD (*p* = 0.0787).

The lack of significant correlations between HbA1c and IL‐35, GAD, or FGF‐18 positive producing cells in the present study can be interpreted in light of evidence that these biomarkers predominantly reflect a localised tissue immune response rather than systemic glycaemic status. In DFU, FGF‐18, IL‐35 and GAD were all markedly elevated in ulcer tissue compared with NDC, while serum FGF‐18 and IL‐35 did not differ significantly between groups, supporting the notion of a compartmentalised immunoinflammatory axis within the wound that is dissociated from circulating markers such as HbA1c. Consistent with this, age, BMI, and diabetes duration also showed no significant associations with tissue FGF‐18, GAD or IL‐35, indicating that these markers are more tightly linked to local wound biology than to general metabolic control. Methodologically, the relatively small number of biopsied patients included in correlation analyses limits statistical power, which the authors acknowledge as a constraint on detecting and generalising associations between biomarkers and clinical variables. Finally, the cytokine milieu in chronic diabetic wounds is highly complex, with multiple feedback loops and unmeasured confounders (e.g., infection status, medication use, overall glycaemic control) that may independently modulate local expression of FGF‐18, IL‐35 and GAD, making a simple linear relationship with HbA1c unlikely and underscoring the need for further studies that better account for these variables.

Cytokines play a crucial role in directing the wound healing process. Researchers may identify potential treatment targets by examining DFU to gain a deeper understanding of how they heal differently from other wounds. Some cytokines can act as biomarkers for the development or recovery of diseases. The cytokine network, however, is complex, with numerous feedback loops and interactions. Due to this intricacy, it may be challenging to predict how treatments targeting specific cytokines will perform. To fully comprehend the role of cytokines in diabetic wound healing and to translate this understanding into effective clinical treatments, further research is still necessary, despite recent advancements.

This study provides novel insights into the expression of key immunological biomarkers, FGF‐18, GAD and IL‐35, in the context of DFU, using both immunohistochemistry and serum analysis. By examining tissue‐level and circulating levels of these markers, the study captures a more comprehensive view of local and systemic inflammatory responses. The use of standardised techniques, such as SPSS v27 for statistical analysis and immunohistochemical quantification per mm^2^, enhances the methodological rigour. Furthermore, the inclusion of both NDC and patients with T2D and DFU allows for relevant clinical comparisons. Employing correlation analyses further allows exploration of associations between biomarker expression and clinical parameters such as age, BMI, and duration of diabetes, adding contextual relevance.

Despite these strengths, several limitations should be acknowledged. The sample size, particularly for the DFU group in the serum analysis, was relatively small, which may limit the statistical power and generalizability of the findings. Additionally, the cross‐sectional design prevents any conclusions about causal relationships between biomarker levels and DFU progression. The absence of longitudinal follow‐up also limits insight into how these biomarkers may vary with wound healing or disease severity over time. Moreover, there was a significant age difference between patients with DFU and NDC, which may introduce residual confounding, even though no significant correlations were observed between age and tissue FGF‐18, GAD or IL‐35 expression in the DFU group. Lastly, although correlations were used, the study does not account for potential confounding variables (e.g., glycaemic control, infection status, medication use), which could influence biomarker expression and should be addressed in future research.

## 5 Conclusion

DFU pathology is driven by a localised, compartmentalised immunoinflammatory axis within the wound tissue, evidenced by the concurrent and highly correlated elevation of FGF‐18, IL‐35 and GAD that is distinct from the patient's systemic circulation. This finding suggests a dysfunctional crosstalk where regenerative signals (FGF‐18) are overwhelmed by chronic inflammation (IL‐35 and GAD), perpetuating the non‐healing state. Consequently, the results strongly support a paradigm shift towards developing highly specific, localised topical therapies to disrupt this particular inflammatory loop and restore proper healing, while also validating these tissue molecules as novel, localised diagnostic or prognostic biomarkers for DFU severity.

## Funding

The authors have nothing to report.

## Ethics Statement

The study was performed in accordance with the World Medical Association's Code of Ethics (Declaration of Helsinki) for studies involving human beings. The research protocol (No. 75, Date: 18 May 2024) was authorised by the Ethical Committee of the Sulaimani University, College of Medicine.

## Consent

Prior to their participation in the study, each subject provided written informed permission. The authors read and approved the final manuscript for publication.

## Conflicts of Interest

The authors declare no conflicts of interest.

## Supporting information


**Figure S1:** Correlation heatmap showing the relationships between FGF‐18, GAD and IL‐35 positive cells per mm^2^ as assessed by immunohistochemistry. Pearson correlation was used to calculate correlation coefficients (*r*), *p* values and 95% confidence intervals (CI). CI, confidence interval; FGF‐18, fibroblast growth factor 18; GAD, glutamic acid decarboxylase; IL‐35, interleukin‐35.
**Figure S2:** Pearson correlation heatmap showing the relationships between HbA1c and the numbers of IL‐35‐, GAD‐ and FGF‐18‐positive producing cells per mm^2^ in patients with diabetic foot lesions. Values within the heatmap cells represent Pearson correlation coefficients (*r*). FGF‐18, fibroblast growth factor 18; GAD, glutamic acid decarboxylase; HbA1c, glycated haemoglobin; IL‐35, interleukin‐35.

## Data Availability

The data that support the findings of this study are available on request from the corresponding author. The data are not publicly available due to privacy or ethical restrictions.
