# Supplementary material for: Immunoinflammatory Profile of FGF‐18, IL‐35 and Glutamic Acid Decarboxylase in Patients With Diabetic Foot Ulcers
Source: Int Wound J. 2026 Jul 3;23(7):e70990. doi: 10.1111/iwj.70990 (PMC13330589; doi:10.1111/iwj.70990)
Supplement: Supplementary file 1 — Figure S1: Correlation heatmap showing the relationships between FGF‐18, GAD and IL‐35 positive cells per mm2 as assessed by immunohistochemistry. Pearson correlation was used to calculate correlation coefficients (r), p values and 95% confidence intervals (CI). CI, confidence interval; FGF‐18, fibroblast growth factor 18; GAD, glutamic acid decarboxylase; IL‐35, interleukin‐35. Figure S2: Pearson correlation heatmap showing the relationships between HbA1c and the numbers of IL‐35‐, GAD‐ and FGF‐18‐positive producing cells per mm2 in patients with diabetic foot lesions. Values within the heatmap cells represent Pearson correlation coefficients (r). FGF‐18, fibroblast growth factor 18; GAD, glutamic acid decarboxylase; HbA1c, glycated haemoglobin; IL‐35, interleukin‐35. [file IWJ-23-e70990-s001.docx]

**DFU Supplementary File**

**
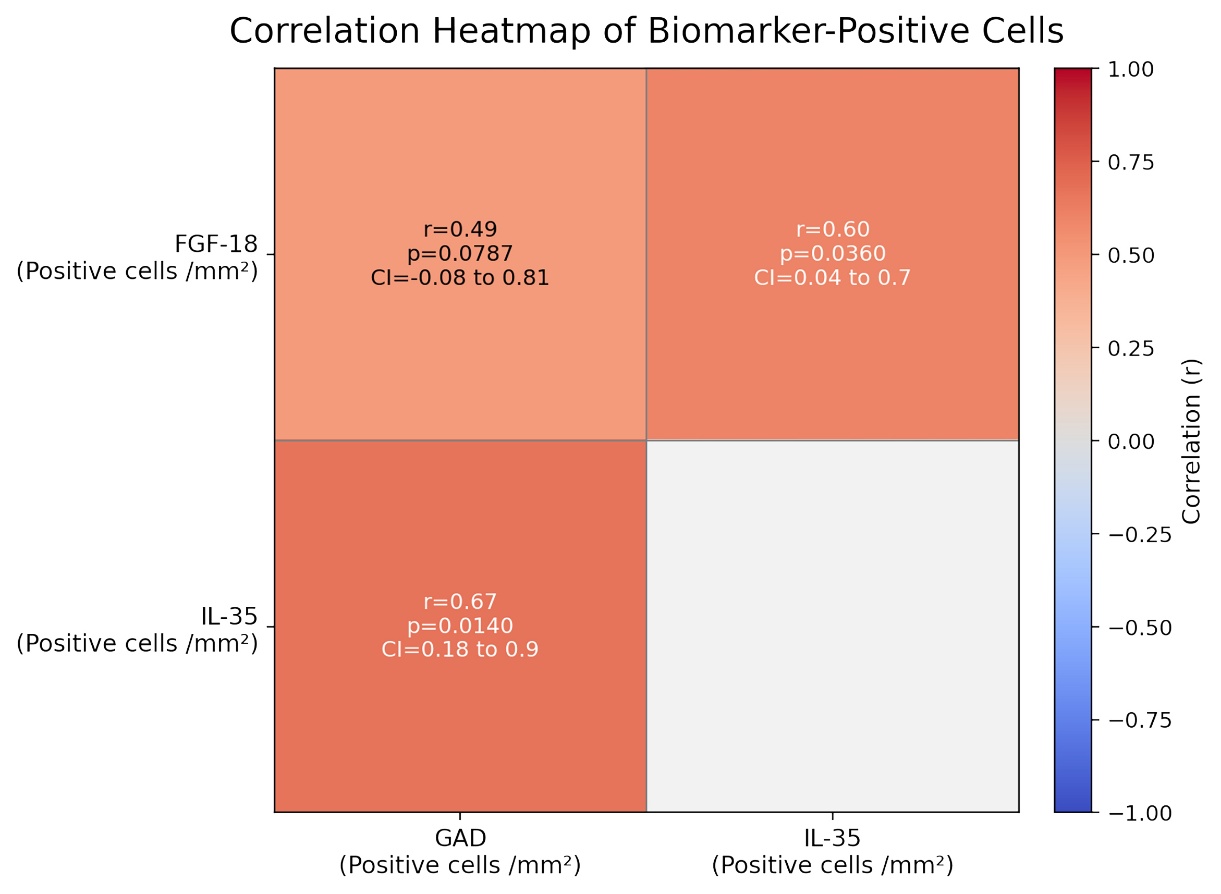
**

**Supplementary Figure 1.** Correlation heatmap showing the relationships between FGF-18, GAD, and IL-35 positive cells per mm² as assessed by immunohistochemistry. Pearson correlation was used to calculate correlation coefficients (r), p-values, and 95% confidence intervals (CI). Abbreviations: GAD, glutamic acid decarboxylase; FGF-18, fibroblast growth factor 18; IL-35, interleukin-35; CI, confidence interval.

**
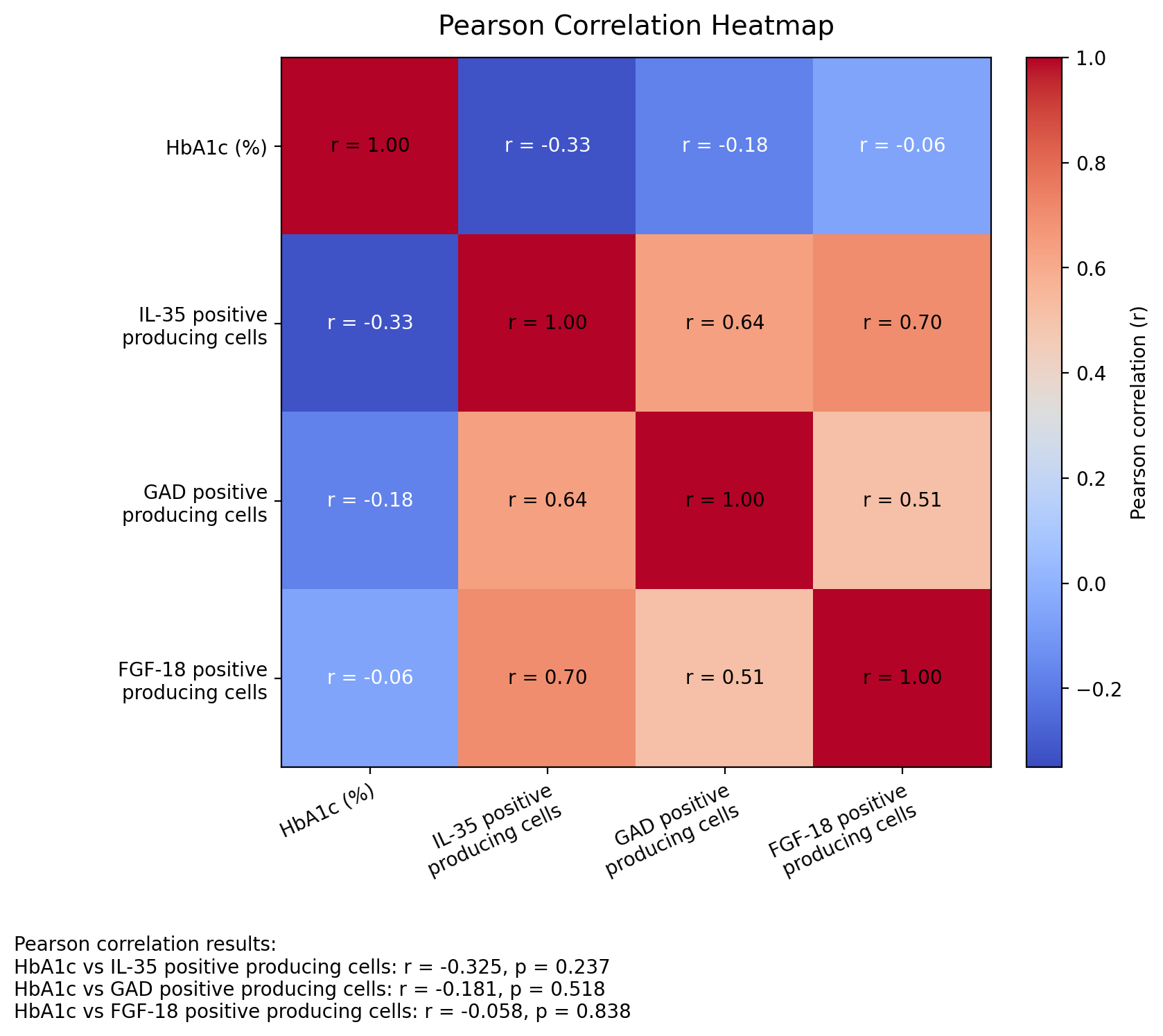
**

**Supplementary Figure 2**. Pearson correlation heatmap showing the relationships between HbA1c and the numbers of IL-35-, GAD-, and FGF-18-positive producing cells per mm² in patients with diabetic foot lesions. Values within the heatmap cells represent Pearson correlation coefficients (r). Abbreviations: HbA1c, glycated hemoglobin; IL-35, interleukin-35; GAD, glutamic acid decarboxylase; FGF-18, fibroblast growth factor 18.
